# Platelet lumiaggregation testing: Reference intervals and the effect of acetylsalicylic acid in healthy adults

**DOI:** 10.5937/jomb0-24690

**Published:** 2020-10-02

**Authors:** Alenka Trampuš-Bakija, Janez Jazbec, Barbara Faganel-Kotnik

**Affiliations:** 1 University Medical Centre Ljubljana (UMC), University Children's Hospital, Institute of Special Laboratory Diagnostic, Ljubljana, Slovenia; 2 University Medical Centre Ljubljana (UMC), University Children's Hospital, Department of Haematology and Oncology, Ljubljana, Slovenia

**Keywords:** blood platelets, platelet aggregation, platelet function test, reference values, referentne vrednosti, test funkcije trombocita, agregacija trombocita, trombociti

## Abstract

**Background:**

Light transmission aggregometry with lumiaggregometry are methods commonly recommended as a first-line test in platelet dysfunction diagnostic work-up. They are poorly standardized and usually performed in specialized laboratories. For proper interpretation, each laboratory should establish its own diagnostic approach in order to recognize abnormal aggregation patterns. The aim of this study was to measure plasma lumiaggregometry with basic agonists to establish the analyzer-reagent reference intervals (RI) for adults and to test the method response to aspirin.

**Methods:**

The Chrono-Log Model 700 lumiaggregometer using Chrono-Par and Chrono-lume reagents (Chrono-Log Corp., Havertown, PA, USA) was used to measure the maximal aggregation and adenosine triphosphate release using adenosine diphosphate (2 μmol/L), collagen (2 μg/mL), arachidonic acid (1 μmol/L), epinephrine (5.5 μmol/L) and ristocetin (1.25 mg/mL), and thrombin (1 U/mL). The effect of aspirin on platelet aggregation and granule release was inspected.

**Results:**

RIs derived from 40 healthy adults were calculated using the non-parametric approach. Wider intervals and low lower limits were determined for weak agonist as well as absence or impaired aggregation in up to one of 7 healthy controls. The response of platelets to aspirin shows response comparable to previously reported study.

**Conclusions:**

Locally established RI in our study enable us to investigate platelet function in patients with a high probability of bleeding disorders. Values are agonist and equipment specific. The variability of the method can be reduced by considering standardized preanalytical and analytical variables. Pathological results must be interpreted in the context of other hemostasis test results and clinical findings.

## Abstract (sr)

**Uvod:**

Optička agregometrija i lumiagregometrija su metode koje se obično preporučuju kao test prvog reda u dijagnostičkoj disfunkciji trombocita. Loše su standardizovani i obično se izvode u specijalizovanim laboratorijama. Za pravilno tumačenje, svaka laboratorija treba da uspostavi svoj dijagnostički pristup kako bi prepoznala abnormalni obrazac agregacije. Cilj ove studije je bio da se izmeri lumiagregometrija u plazmi sa osnovnim agonistima kako bi se utvrdio referentni interval reagensa i analizatora (RI) kod odraslih osoba i testirala reakcija metode na aspirin.

**Metode:**

Korišćen je lumiagregometar Chrono-Log Model 700 koji koristi Chrono-Par i Chrono-lume reagense (Chrono-Log Corp., Havertown, Pensilvanija, SAD) za merenje maksimalne agregacije i oslobađanja adenozin-trifosfata korišćenjem adenozin-difosfata (2 μmol/L), kolagena (2 μg/mL), arahidonske kiseline (1 mmol/L), epinefrina (5,5 μmol/L), ristocetina (1,25 mg/mL) i trombina (1 U/mL). Ispitan je uticaj aspirina na agregaciju trombocita i oslobađanje granula.

**Rezultati:**

Neparametarskim pristupom je izračunat RIdobijen od 40 zdravih odraslih osoba. Za slabe agoniste suodre|eni širi intervali sa nižim donjim granicama. Oslabljena agregacija ili njeno odsustvo je pronađeno kod najviše jedne od sedam zdravih osoba iz kontrolne grupe. Reakcija trombocita na aspirin je uporediva sa ranije objavljenim ispitivanjima.

## Introduction

The evaluation of platelet function by light transmission aggregation (LTA) or lumiaggregation is a gold standard for patients with platelet function defects. Both assays are time-consuming and technically challenging. In spite of that, the method is recommended as a first-line test in platelet dysfunction diagnostic workup [1]. Abnormal bleeding, as a consequence of altered platelet generation or activation, may lead to a severe clinical condition. The discrimination between inherited and acquired conditions is a difficult task for clinicians but crucial for adequate management of patients. Acquired conditions are more frequent, while the incidence of inherited platelet dysfunction is ranging from 1/1.000.000 to 1/100 for some mild defects [2]. In both types, the examination of clinical history is followed by preliminary laboratory tests: peripheral blood platelet count with smear examination, activated thromboplastin time, prothrombin time, thrombin time, fibrinogen concentration, and von Willebrand disease screening. Those methods are well standardized and worldwide accessible. Most second and third step tests (i.e. LTA, granule release, flow cytometry, electron microscopy) are much less standardized, and usually they are performed in specialized laboratories. Among them, LTA is the most commonly performed method, followed by platelet dense granule release test in smaller proportions [3] [4]. Guideline documents for LTA are not uniform about the very best practice in performing and interpreting test results [5] [6] [7] [8]. For proper interpretation, each laboratory should establish its own approach to the diagnosis and cut-off values for distinguishing abnormal findings from a normal aggregation pattern. Rare genetic defects in platelet membrane proteins (i.e. Glanzmann thrombasthenia) have reproducible and characteristic profiles and are easily recognized. In addition to standard LTA method, simultaneous measurement of platelet secretion could be of greater value with inherited defect of platelet function as it shows greater sensitivity to much common mild disorders of platelet secretion [9] [10] [11]. The aggregation response to antiplatelet agents (e. g. aspirin, clopidogrel) is well described but the monitoring with classical LTA is discouraged in clinical practice [8] [9]. Several points of care tests (VerifyNow, Multiplate Electrode Aggregometry, Platelet function analyzer) have become available to monitor antiplatelet therapy. In spite of much evidence on diagnostic usefulness of (lumi)aggregometry, recent studies questioned the practical utility of dense granule release as the assay variability (especially for ATP release) is relatively high (CV>20%) [12].

There are only four specialized laboratories in Slovenia where LTA can be performed and the laboratory at Children's hospital Ljubljana is the only one where lumiaggregometry for platelet function diagnostic workup is routinely done. The aim of the study was to measure plasma LTA and ATP secretion with several agonists to establish the analyzer-reagent reference intervals for adults. In addition, the utility of the test was validated in subjects receiving aspirin.

## Materials and Methods

### Subjects

Blood was collected from 45 healthy subjects, 26 females and 19 men, aged from 24 to 63. Exclusion criteria for volunteers were the absence of acute disease, absence of any medical therapy for 14 days, pregnancy and known coagulation disorder. Samples were collected from 8.00 to 10.00 am during two months period.

For acetylsalicylic acid effect monitoring, 11 volunteers from the above described same group of healthy subjects received aspirin in a single dose of 500 mg (if body weight was up to 70 kg) or 1000 mg (if body weight was above 70 kg) 24 hours prior to venepuncture.

Written study instructions were given, and informed consent was obtained from all participants. The study was approved by the National Medical Ethics Committee of the Republic of Slovenia (approval No 53/08/11) in accordance with the Helsinki Declaration.

### Methods

Blood samples were collected into 0.109 mol/L buffered sodium citrate tubes (2x4.5 mL Becton Dickinson, Franklin Lakes, NJ, USA). The samples were processed within a one-hour interval, and analysis was completed within four hours after blood collection.

Platelet-rich plasma (PRP) was prepared by centrifugation at 200×g for 10 minutes at room temperature. Plasma from both collection tubes was transferred into a polypropylene tube and gently mixed. PRP was then adjusted to a count from 200 to 300×10^9^ platelets/L, using autologous platelet-poor plasma (PPP) (2500×g, 15 min). PRP was capped and kept at room temperature until further analyses. PRP with platelet count lower than 200×10^9^ /L was excluded from experiments. Platelet count in plasma was determined by AcT 8 hematology analyzer (Beckman Coulter, Fullerton, CA, USA).

Analyses were performed on lumiaggregometer Chrono-Log Model 700 (Chrono-Log Corp., Havertown, PA, USA) and data processed with software program Aggro/link 8 (Chrono-Log, Havertown, PA, USA).

The following Chrono-par® and Chronolume® reagents (all Chrono-Log, Havertown, PA, USA) in final concentrations in PRP were used in LTA and lumiaggregometry experiments: ADP (2 μmol/L), collagen (2 μg/mL), arachidonic acid (1 mmol/L), epine phrine (5.5 μmol/L), ristocetin (1.25 mg/mL), thrombin (1 U/mL) and ATP standard (2 nmol). For lumiaggregometry we used luciferin-luciferase reagent (0.2 μmol/L).

ATP release was tested according to manufacturer instrument instructions using: 450 μL of preincubated (37 °C, 3 min) PRP, 50 μL of luciferase reagent, and 10-50 μL of agonist. The aggregation and ATP release were monitored simultaneously at least 5 minutes after adding an agonist or 10 minutes if maximal aggregation was not achieved within 5 minutes [8]. Results were reported as maximum aggregation (%) and concentration of released ATP (nmol/L).

If the limited amount of sample was available, the reaction was performed with 225 μL of plasma and adjusted volumes of all reagents.

### Statistical analysis

Reference intervals (95%, double-sided), ranging from 2.5 to 97.5 percentiles, were calculated using the non-parametric percentile method (CLSI C28-A3 guidelines) [13] since the distributions were normal after one elimination of suspected outliers (Turkey calculation). The statistical software Med -Calc ® (version 11.3.0.0., Frank Schoonjans, Mariakerke, Belgium) was used for analysis.

## Results

Reference intervals for percentage of maximal aggregation for selected agonists are summarized in (Table 1). The number of control subjects for each agonist ranged from 38 to 44. One set of values was used for each collected sample; some data have been excluded either due to low platelet count or as outliers. D'Agostino Pearson test indicates normal distribution for all agonists after the removal of outliers. Few results with no response (zero % of aggregation) were observed for week agonist stimulation, and those were also excluded from calculation. The lowest lower reference limit was observed with ADP, while for other agonists values above 60% of maximal aggregation were observed. Thrombin, arachidonic acid, and collagen had highest lower RI limit for ATP release. In the sense of statistics, 0-4.7% of values of healthy control subjects (0-2 results in the control population) exceeded the reference interval. The median platelet count in PRP was 274 x 10^9^ /L (range: 163-302 x 10^9^ /L).

**Table 1:** Light transmission aggregation and ATP release reference intervals for adults. Statistical methods included nonparametric analyses. ADP – adenosine diphosphate; ATP – adenosine triphosphate; NA – not assayed

Agonist	Final concentration	Reference intervals (n=number of sample)	
		% maximal aggregation	ATP concentration (nmol)
ADP	2 μmol/L	55–87 (n=44)	0.29–2.11 (n=42)
Epinephrine	5.5 μmol/L	63–92 (n=38)	0.22–2.60 (n=38)
Collagen	2 μg/mL	65–92 (n=40)	0.41–2.33 (n=39)
Arachidonic acid	1 mmol/L	62–85 (n=41)	0.49–2.46 (n=38)
Ristocetin	1.25 mg/mL	62 – 91 (n=43)	NA
Thrombin	1 U/mL	NA	0.50–2.85 (n=42)

The effect of aspirin therapy on maximal aggregation and ATP release was tested on 11 healthy volunteers receiving acetylsalicylic acid before venipuncture. The response to selected agonists is described in (Table 2). In majority of samples, impaired aggregation to all agonists except for ristocetin and low ATP release with the exception of thrombin and collagen were determined. The assay showed 100% sensitivity for aggregation response to epinephrine 5.5 μmol/L, collagen 2 μmol/mL and arachidonic acid 1 mmol/L as well as for ATP release level induced with ADP 2 μmol/L, epinephrine 5.5 μmol/L and arachidonic acid 1 mmol/L.

**Table 2:** Light transmission aggregation and ATP release level in subjects receiving acetylsalicylic acid. The table presents average responses to agonists in comparison to the lower reference interval limit. Arachidonic acid and epinephrine show maximum sensitivity to acetylsalicylic acid. RI – reference interval; NA – not assayed; ADP – adenosine diphosphate; ATP – adenosine triphosphate

Agonist (final concentration)	Lower RI limit % maximal aggregation	Average % maximal aggregation (No. of reduced responses/total)	Lower RI limit ATP concentration (nmol)	Average ATP concentration (No. of reduced responses/total)
ADP (2 μmol/L)	55	51(6/11)	0.29	0.0(11/11)
Epinephrine (5.5 μmol/L)	63	19(11/11)	0.22	0.0(11/11)
Collagen (2 μg/mL)	65	36(11/11)	0.41	0.50(6/11)
Arachidonic acid (1 mmol/L)	62	5(11/11)	0.49	0.0(11/11)
Ristocetin (1.25 mg/mL)	62	70(2/11)	NA	NA
Thrombin (1 U/mL)	NA	NA	0.50	1.04(2/11)

## Discussion

The primary goal of our study was to establish a reference interval for maximal aggregation and ATP release in adults, measured by the lumiaggregometric method. Published RIs are usually not transferable and should be established in the local laboratory [13]. The standardization of the aggregometry methods is still lacking and is under investigation. As many as possible preanalytical and analytical variables need to be standardized to assure low variability of the results. In our study, we considered recommendation from different guidelines and evidence-based practices. Variability was reduced by taking fasting sample, venipuncture by the same experienced phlebotomist, time of sampling (before 10 am), time to process the sample (within one hour), time to analyze the sample (within four hours), usage of the same trademark of blood collection material, adjusting PRP count to 200-300 x 10^9^ platelets/L, capping tubes before analysis, resting platelets after centrifugation (at least 30 minutes), performing test at constant room temperature (21–25 °C), using same laboratory equipment and assaying the tests by the same technician. Hemolyzed, lipemic, coagulated, thrombocytopenic samples or underfilled tubes were not analyzed. We used the basic agonist panel and concentrations as it is recommended by international, European and North American guidelines [6] [7] [8]. Low to moderate concentrations of ADP, epinephrine and collagen were chosen to detect the δ-storage pool deficiency.

When method verification is performed, standard reference material could be of great importance. Unfortunately, in the case of platelet function tests, there is no such material available, so the traceability of analytical system is not possible. Therefore, the procedure cannot be performed to the same extent as with more standardized tests for hemostasis. The challenge to establish reference interval is even higher when the analysis must be performed on fresh blood samples when several preanalytical influences could be present. Moreover, last but not least is the cost of the analysis which is considerable. Practices from external quality control schemes show that less than half of participants validate RI, including a low number of healthy volunteers (reported by Hayward on ISLH meeting; Vancouver 2019).

In our study, approximately 40 subjects were included in each agonist group. The group is too small for using the parametric statistical approach; therefore, we used a non-parametrical method to establish intervals as recommended by guidelines of British Society for Hematology [7]. This approach is also suitable for the analysis of not normally distributed data. The sample size below 120 cannot provide coverage probabilities so the confidence intervals for low in high limits could not be calculated. We reported the intervals with lower and upper limits, though values below lower limits are clinically relevant for diagnosis of platelet function disorder. The shape of the curve is subjectively evaluated concomitantly.

The comparison of our findings with the existing publications is difficult due to usage of different equipment, reagents, concentrations of agonists and statistical approach. The manufacturer (Chrono-Log) normal ranges were obtained from various publications and serve as a guideline only. Similar to our findings, the lowest minimum aggregation response with ADP and minimum ATP release with epinephrine (mean±1 SD) was stated. In general, majority of researchers reported broad reference intervals for week agonists (ADP, epinephrine) and narrow ones for strong agonists (arachidonic acid, ristocetin) induced aggregation/agglutination [11] [14] [15] [16]. Some studies reported very low values for lower RI limit when using week agonists. Absence or impaired aggregation is found in up to one of seven healthy controls [11] [14] [16] [17]. In our study, we found absent or reduced response for epinephrine and ADP after 10 minutes of recording in five and two healthy subjects, respectively. These results were excluded from the statistical calculation. The interpretation of per se pathological results when subject's bleeding history is negative is not considered abnormal [18]. The heterogeneity of published RI for ATP release is even broader. It is well known that the variability for ATP release assay is up to 30% and that the test has high specificity and moderate sensitivity [19] [20] [21]. This fact is recently raising question about the usefulness of the assay [4].

Further, examination of primary hemostasis and platelet function in children is challenging and poorly investigated. Pediatric RI is hard to establish due to ethical issues. According to published data [15] [22] [23] [24], RI for adults can be used for the interpretation of results in children older than one year, and this practice was accepted in our laboratory as well. North American Specialized Coagulation Laboratory Association - NASCOLA consensus agreement is even less strict and the usage of LTA RI for adults and children beyond neonatal period is recommended [6].

The limitation of the present study is the number of healthy volunteers to assure the sufficient statistical power of the RI calculation (less than 120). Also, verification of the test's precision was not possible. Multiple sampling of the same patients is difficult to perform and no standardized material has been available so far. If possible, a healthy control subject's sample should be run in parallel with a patient's sample. The reported RI is not validated for PRP with low platelet count. Derived limits and agonist panel were recommended for thrombocytopenic patients [8] [14]. In our study, the response of platelets to acetylsalicylic acid was monitored in order to verify the usefulness of the test and not for the clinical interpretation of antiplatelet therapy. The characteristic absent aggregation and ATP release due to absent cyclooxygenase activity were found in arachidonic acid and epinephrine induced activation. ADP and collagen lower sensitivity is in agreement with previously reported studies showing a very good overall sensitivity of a method to aspirin-like defects [25]. In addition, to confirm an aspirin-like defect (drug-induced or inherited) it would be reasonable to include a thromboxane analogue in the agonist panel.

The above presented locally established RI enables us to investigate platelet function and granule release/storage abnormalities by measuring the LTA and ATP release in patients with high probability of platelet disorders. In case of possible false-positive results, the test should be repeated another day with higher agonist concentrations. Pathological results should always be interpreted in the context of clinical findings. Our results provide RI that could be used or validated by other laboratories using similar local technical conditions in order to interpret results accordingly.

*Acknowledgments*. The authors thank Mojca Juretič for technical support.

## Conflict of interest statement

The authors state that they have no conflicts of interest regarding the publication of this article.

## List of abbreviations

ADP, adenosine diphosphate; ATP, adenosine triphosphate; CV, coefficient of variation; LTA, light transmission aggregation; NA, not assayed; PPP, platelet- poorplasma; PRP, platelet-rich plasma; RI, reference interval; SD, standard deviation

## References

[1] Gresele P, International Society on Thrombosis and Hemostasis Subcommittee on Platelet Physiology (2015). Diagnosis of inherited platelet function disorders: Guidance from the SSC of the ISTH. J Thromb Haemost.

[2] Gresele P, Falcinelli E, Bury L (2018). Laboratory diagnosis of clinically relevant platelet function disorders. Int J Lab Hematol.

[3] Hayward C P M, Moffat K A, Plumhoff E, Timleck M, Hoffman S, Spitzer E, van Cott E, Meijer P (2012). External quality assessment of platelet disorder investigations: Results of international surveys on diagnostic tests for dense granule deficiency and platelet aggregometry interpretation. Semin Thromb Hemost.

[4] Hayward C P M, Moffat K A, Brunet J, Carlino S A, Plumhoff E, Meijer P, Zehnder J L (2019). Update on diagnostic testing for platelet function disorders: What is practical and useful?. Int J Lab Hematol.

[5] Clinical and Laboratory Standards Institute (CLSI) (2008). Platelet function testing by aggregometry: Approved guideline. CLSI document H58-A.

[6] Hayward C P M, Moffat K A, Raby A, Israels S, Plumhoff E, Flynn G, Zehnder J L (2010). Development of North American consensus guidelines for medical laboratories that perform and interpret platelet function testing using light transmission aggregometry. Am J Clin Pathol.

[7] Harrison P, Mackie I, Mumford A, Briggs C, Liesner R, Winter M, Machin S (2011). Guidelines for the laboratory investigation of heritable disorders of platelet function. Br J Haematol.

[8] Cattaneo M, Cerletti C, Harrison P, Hayward C P M, Kenny D, Nugent D, Nurden P, Rao A K, Schmaier A H, Watson S P, Lussana F, Pugliano M T, Michelson A D (2013). Recommendations for the standardization of light transmission aggregometry: A consensus of the working party from the platelet physiology subcommittee of SSC/ISTH. J Thromb Haemost.

[9] Cattaneo M (2009). Light transmission aggregometry and ATP release for the diagnostic assessment of platelet function. Semin Thromb Hemost.

[10] Nieuwenhuis H K, Akkerman J W, Sixma J J (1987). Patients with a prolonged bleeding time and normal aggregation tests may have storage pool deficiency: Studies on one hundred six patients. Blood.

[11] Quiroga T, Goycoolea M, Matus V, Zúñiga P, Martínez C, Garrido M, Aranda E, Leighton F, Panes O, Pereira J, Mezzano D (2009). Diagnosis of mild platelet function disorders: Reliability and usefulness of light transmission platelet aggregation and serotonin secretion assays. Br J Haematol.

[12] Badin M S, Graf L, Iyer J K, Moffat K A, Seecharan J L, Hayward C P M (2016). Variability in platelet dense granule adenosine triphosphate release findings amongst patients tested multiple times as part of an assessment for a bleeding disorder. Int J Lab Hematol.

[13] Clinical and Laboratory Standard Institute (CLSI) (2008). Define and verify reference intervals in the clinical laboratory: Approved guideline.

[14] Hayward C P M, Moffat K A, Pai M, Liu Y, Seecharan J, Mckay H, i dr. (2008). An evaluation of methods for determining reference intervals for light transmission platelet aggregation tests on samples with normal or reduced platelet counts. Thromb Haemost.

[15] Knöfler R, Streif W (2010). Strategies in clinical and laboratory diagnosis of inherited platelet function disorders in children. Transfus Med Hemother.

[16] Platton S, Mccormick Á, Bukht M, Gurney D, Holding I, Moore G W (2018). A multicenter study to evaluate automated platelet aggregometry on Sysmex CS-series coagulation analyzers: Preliminary findings. Res Pract Thromb Haemost.

[17] Mezzano D, Quiroga T, Pereira J (2009). The level of laboratory testing required for diagnosis or exclusion of a platelet function disorder using platelet aggregation and secretion assays. Semin Thromb Hemost.

[18] Zhou L, Schmaier A H (2005). Platelet aggregation testing in platelet-rich plasma: Description of procedures with the aim to develop standards in the field. Am J Clin Pathol.

[19] Pai M, Wang G, Moffat K A, Liu Y, Seecharan J, Webert K, Heddle N, Hayward C (2011). Diagnostic usefulness of a lumi-aggregometer adenosine triphosphate release assay for the assessment of platelet function disorders. Am J Clin Pathol.

[20] Brunet J G, Iyer J K, Badin M S, Graf L, Moffat K A, Timleck M, Spitzer E, Hayward C P M (2018). Electron microscopy examination of platelet whole mount preparations to quantitate platelet dense granule numbers: Implications for diagnosing suspected platelet function disorders due to dense granule deficiency. Int J Lab Hematol.

[21] Castilloux J F, Moffat K A, Liu Y, Seecharan J, Pai M, Hayward C P M (2011). A prospective cohort study of light transmission platelet aggregometry for bleeding disorders: Is testing native platelet-rich plasma non-inferior to testing platelet count adjusted samples?. Thromb Haemost.

[22] Bonduel M, Frontroth J P, Hepner M, Sciuccati G, Feliú-Torres A (2007). Platelet aggregation and adenosine triphosphate release values in children and adults. J Thromb Haemost.

[23] Hvas A, Favaloro E J (2017). Platelet function testing in pediatric patients. Expert Rev Hematol.

[24] Ravn H B, Andreasen J B, Hvas A (2017). Does whole blood coagulation analysis reflect developmental haemostasis?. Blood Coagul Fibrinolysis.

[25] Taylor M l, Misso N L A, Stewart G A, Thompson P J (1992). The effect of varying doses of aspirin on human platelet activation induced by PAF, collagen and arachidonic acid. Br J Clin Pharmacol.

